# Regulation of Sulfur Homeostasis in Mycorrhizal Maize Plants Grown in a Fe-Limited Environment

**DOI:** 10.3390/ijms21093249

**Published:** 2020-05-04

**Authors:** Styliani N. Chorianopoulou, Petros P. Sigalas, Niki Tsoutsoura, Anastasia Apodiakou, Georgios Saridis, Yannis E. Ventouris, Dimitris L. Bouranis

**Affiliations:** 1 Plant Physiology and Morphology Laboratory, Crop Science Department, Agricultural University of Athens, 75 Iera Odos, Athens 11855, Greece; yannisventouris@gmail.com (Y.E.V.); bouranis@aua.gr (D.L.B.); 2 Plant Science Department, Rothamsted Research, West Common, Harpenden, Hertfordshire AL5 2JQ, UK; petros.sigalas@rothamsted.ac.uk; 3 School of Biosciences, Faculty of Science, University of Nottingham, Sutton Bonington Campus, Sutton Bonington, Leicestershire LE12 5RD, UK; nikitsoutsoura@gmail.com; 4 Max Planck Institute of Molecular Plant Physiology, 14476 Potsdam Golm, Germany; apodiakou@mpimp-golm.mpg.de; 5 Botanical Institute, Cologne Biocenter, University of Cologne, D–50674 Cologne, Germany; g.saridis@uni-koeln.de

**Keywords:** iron limitation, maize, mycorrhizal symbiosis, sulfate assimilation, sulfate transporters, sulfate uptake, sulfur deprivation

## Abstract

Sulfur is an essential macronutrient for growth of higher plants. The entry of the sulfate anion into the plant, its importation into the plastids for assimilation, its long-distance transport through the vasculature, and its storage in the vacuoles require specific sulfate transporter proteins. In this study, mycorrhizal and non-mycorrhizal maize plants were grown for 60 days in an S-deprived substrate, whilst iron was provided to the plants in the sparingly soluble form of FePO_4_. On day 60, sulfate was provided to the plants. The gene expression patterns of a number of sulfate transporters as well as sulfate assimilation enzymes were studied in leaves and roots of maize plants, both before as well as after sulfate supply. Prolonged sulfur deprivation resulted in a more or less uniform response of the genes’ expressions in the roots of non-mycorrhizal and mycorrhizal plants. This was not the case neither in the roots and leaves after the supply of sulfur, nor in the leaves of the plants during the S-deprived period of time. It is concluded that mycorrhizal symbiosis modified plant demands for reduced sulfur, regulating accordingly the uptake, distribution, and assimilation of the sulfate anion.

## 1. Introduction

Sulfur is an essential macronutrient for growth of higher plants. Plants take up sulfate mainly from the soil solution in order to synthesize essential organic sulfur compounds. It composes a part of proteins in the form of cysteine and methionine. Apart from amino acids, sulfur is present in a large number of co-enzymes, prosthetic groups, and secondary metabolites [1,2]. The entry of the sulfate anion into the plant, its importation into the plastids for assimilation, its long-distance transport through the vasculature, and its storage in the vacuoles require specific sulfate transporter proteins [1,2,3]. The influx of sulfate occurs against the inside-negative gradient of the membrane potential, requiring a driving force for transport. In this line, sulfate transporters are pH-dependent proton/sulfate co-transporters containing 10–12 membrane-spanning helices. In *Arabidopsis thaliana*, sulfate transporters have been classified into 4 groups according to their phylogenetic relationships, tissue-specific expression, and kinetic properties; twelve proteins have been characterized in total. Sulfate transporters belonging to group 1 are high-affinity transporters; they are localized in the root and serve such functions as sulfate uptake from the soil solution [4,5,6,7]. Sulfate transporters belonging to group 2 assist in sulfate transport from root to shoot and especially they absorb sulfate from the apoplast and are considered low-affinity transporters [4,6]. Group 3 of sulfate transporters has been described as a leaf-expressed group which participates in sulfate transport into plastids, specifically in the chloroplast [8,9]. Sulfate transporters belonging to group 4 are localized in the tonoplast and facilitate the sulfate efflux from the vacuoles [2,3,10].

Once sulfate enters the cell, it can either enter the assimilation pathway, which occurs in both plastids and cytosol, or be stored in the vacuole [11]. Enzyme adenosine 5’-phosphosulfate reductase (APR) is considered to be a key regulatory point in the plastidic sulfate assimilation and reduction, because it plays a crucial role in sulfur partitioning between the primary and secondary metabolism and coordinates S, N, and C assimilation [12]. More specifically, APR is an iron–sulfur protein which catalyzes the two-electron reduction of adenosine 5’-phosphosulfate (APS) to sulfite using glutathione as an electron donor. The reduction of sulfite to sulfide is catalyzed by a plastid-localizing sulfite reductase (SiR), which uses ferredoxin as a reductant. Sulfide generated in plastids is the substrate for cysteine biosynthesis [2].

Sulfate uptake and assimilation are tightly regulated according to the plant demands for reduced sulfur. Upon sulfate starvation, plants prioritize the biosynthesis of reduced sulfur compounds and the control of sulfur assimilation occurs primarily at the steps of sulfate uptake and APS reduction. The metabolic pathway from the uptake and reduction of sulfate to cysteine biosynthesis is therefore the basis of sulfur metabolism. It is suggested that sulfate uptake and APS reduction are the most highly regulated steps of the pathway [13]. Additionally, there is strong evidence that the regulation of sulfate uptake and assimilation is well-correlated with regulation of mRNA levels of the corresponding genes. In this line, transcriptional regulation of *Arabidopsis* SULTR1.1, 1.2, 2.1, 4.1, 4.2, and APR have been monitored under sulfate starvation. The transcript levels of sulfate transporters (SULTR) are rapidly reduced when sulfate is resupplied to sulfur-starved plants [14]. APR is the key regulatory enzyme in the sulfate assimilation pathway taking place in plastids. In *Arabidopsis thaliana*, the transcript and the protein levels of the APR isoenzymes increase in response to sulfate starvation, while they decrease under sulfate-sufficient conditions [15]. In other words, the expression of APR is highly regulated in a demand-driven manner [16].

Maize follows strategy II for iron uptake, a strategy that requires the biosynthesis of deoxymugineic acid (DMA) for efficient ferric iron chelation and acquisition from the rhizosphere. The primary precursor of DMA is sulfur-containing amino acid methionine, and this is the reason why S homeostasis has a strong effect on iron homeostasis. In a previous study, it was depicted that arbuscular mycorrhizal symbiosis altered the expression patterns of iron homeostasis genes in S-deprived maize plants [17]. More specifically, mycorrhizal symbiosis prevented Fe deprivation responses in S-deprived maize plants. Mycorrhizal plants responded as if they sensed Fe-efficient conditions, whilst non-mycorrhizal plants were at Fe-deficient conditions throughout the treatment, and it was hypothesized that iron was possibly provided to mycorrhizal plants through a symbiotic pathway. Both mycorrhizal and non-mycorrhizal plants needed to produce DMA in order to chelate the insoluble ferric iron from their rhizosphere. This need was greater for the non-mycorrhizal plants and it was prominent both before as well after sulfur supply. In this study, the same system was used in order to investigate the response of the sulfur uptake and assimilation pathway to those needs and Fe limitation was used to stimulate sulfate uptake and assimilation for DMA biosynthesis. How did the plants coordinate their sulfur homeostasis to meet their needs for DMA biosynthesis? In order to address this question, the putative sulfate transporters as well as the corresponding sulfate assimilation enzymes were identified via an in silico analysis and their expression patterns were studied in leaves and roots of mycorrhizal and non-mycorrhizal maize plants growing in a Fe-limited medium before and after sulfate supply.

## 2. Results

### 2.1. In Silico Analysis

**Figure 1:**
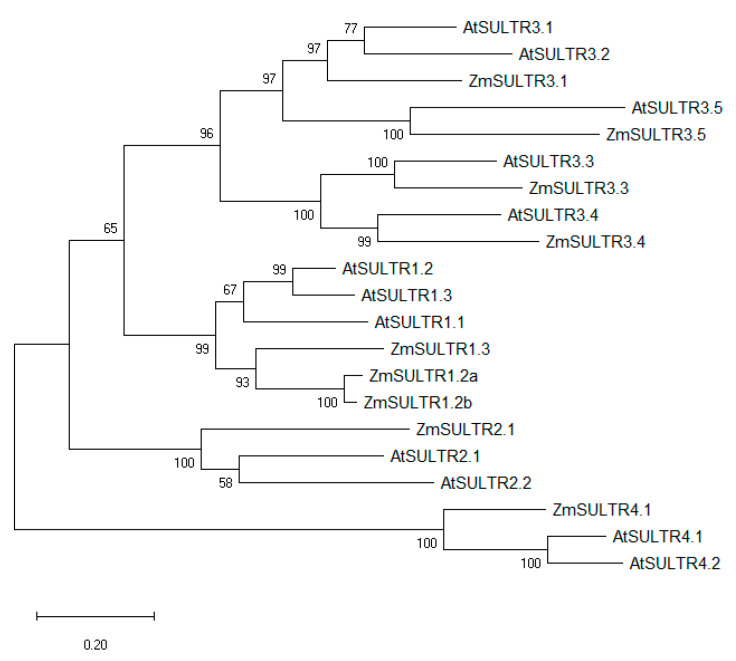
Evolutionary analysis by the maximum likelihood method among the protein sequences of the sulfate transporters of *Arabidopsis thaliana* and *Zea mays*. The evolutionary history was inferred by using the maximum likelihood method and the Jones-Taylor-Thornton (JTT) matrix-based model [19]. Evolutionary analyses were conducted in MEGA X [20].

Bioinformatic analysis of data from public databases (NCBI: https://www.ncbi.nlm.nih.gov/, and Maize Genome Database: https://www.maizegdb.org/ [18]) has revealed 9 putative members in the maize sulfate transporter family (Table 1). The protein sequences of the sulfate transporters of *Arabidopsis thaliana* were utilized to identify the maize homologs through protein BLAST (Basic Local Alignment Search Tool) in the Maize Genome Database. Phylogenetic comparison of the protein sequences discovered in maize to those of the *Arabidopsis thaliana* sulfate transporters has revealed that the maize putative sulfate transporter homologs (ZmSULTR) correspond well to the known classifications in other species and can be sorted into 4 distinct groups (Figure 1). Group 1 contains ZmSULTR1.2a, ZmSULTR1.2b, and ZmSULTR1.3. Group 2 and group 4 are single-membered and consist of ZmSULTR2.2 and ZmSULTR4.1, respectively. On the other hand, group 3 can be further divided into two subgroups: the first includes transporters ZmSULTR3.1 and ZmSULTR3.5, and the other is comprised of ZmSULTR3.3 and ZmSULTR3.4. Maize sulfate transporter nomenclature was based on sequence similarities to sulfate transporters of *Arabidopsis thaliana* as determined by the phylogenetic tree (Figure 1). Interestingly, maize does not appear to possess a SULTR1.1 homolog. Protein BLAST (NCBI) has revealed that both ZmSULTR1.2a and ZmSULTR1.2b share a higher degree of similarity to AtSULTR1.2 than to AtSULTR1.1. Thus, it is possible that an ancestral SULTR1.2 underwent duplication in maize at some point after the separation of monocots and dicots. Similarly, the protein sequences of AtAPRL1, AtAPRL2, and AtSiR led to the unravelling of their homologs in maize.

RNA-Seq data from the Maize Genome Database regarding the expression patterns of sulfate transporters and sulfur assimilation genes imply that transcripts of all *ZmSULTR* genes are found both in roots and leaves with the exception of these of *ZmSULTR1.3* and *ZmSULTR3.3* which are absent from roots, and this of *ZmSULTR1.2a* which is not present in leaves. On the other hand, *ZmSULTR3.4* displays low levels of expression in roots and leaves, whereas *ZmSULTR1.2b* lacks expression throughout the plant body. According to this data series, *ZmAPRL1*, *ZmAPRL2*, and *ZmSiR* transcripts are detected both in roots and leaves. By taking into account this information, a more efficient planning of gene expression analysis under S-sufficient, S-deficient conditions, as well as after S resupply in mycorrhizal (M) and non-mycorrhizal (NM) plants was possible.

### 2.2. Sulfate Uptake and Transport in Roots

**Figure 2:**
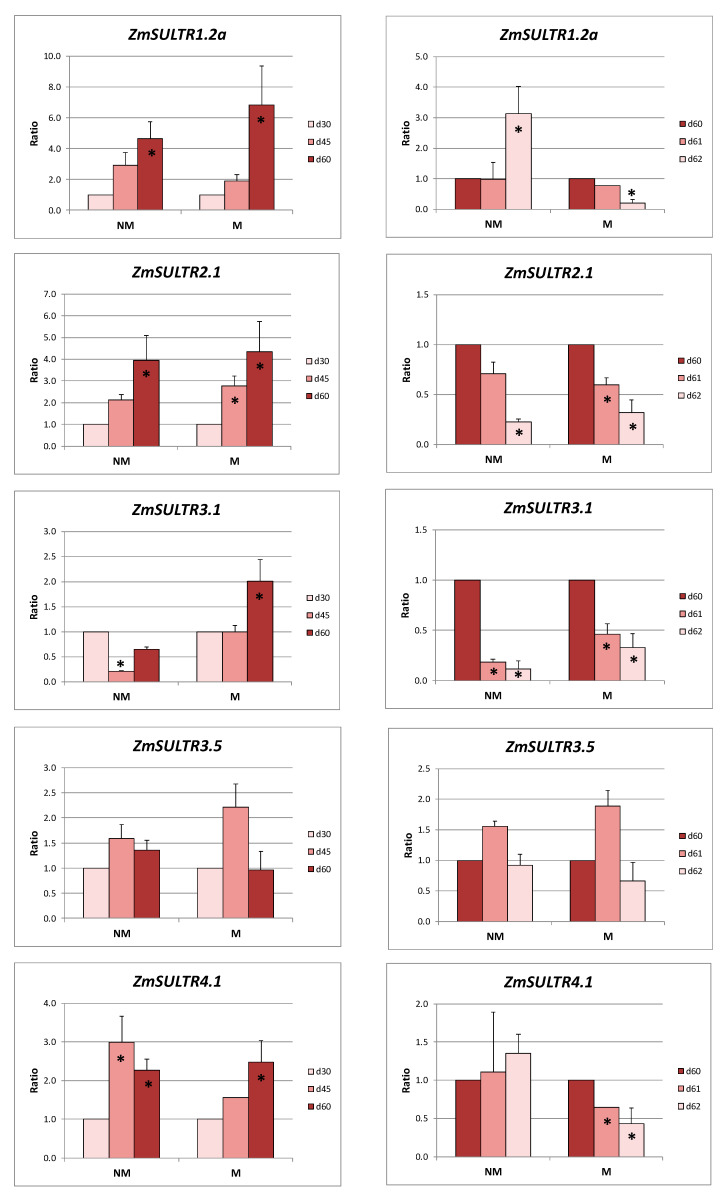
Relative expression ratios (the mean of six biological replicates ± standard error (SE)) of the sulfate transporters in the roots of non-mycorrhizal (NM) and mycorrhizal (M) maize plants before (left column) and after (right column) the supply of sulfate. d30/d45/d60/d61/d62: days after sowing. Significant differences (*p* < 0.05) between the samples and the respective controls are represented by an asterisk (*).

The long-term sulfur starvation resulted in a strong overexpression of *ZmSULTR1.2a*, *ZmSULTR2.1*, and *ZmSULTR4.1* in the roots of NM plants. On the other hand, *ZmSULTR3.1* was downregulated during the same period of time. The roots of M plants presented the same behavior regarding the overexpression of the first three genes, whilst the expression levels of *ZmSULTR3.1* remained constant (Figure 2).

The addition of sulfate resulted in differentiated responses in the expression of various genes in NM and M plants: *ZmSULTR1.2a* in the roots of NM plants was the only gene which was upregulated, *ZmSULTR4.1* in the roots of NM plants was the only gene with stable expression levels, whilst in all other genes and cases a strong downregulation was observed. No statistically significant changes in the expression levels of *ZmSULTR3.5* were observed neither before nor after sulfate supply in NM and M plants (Figure 2).

Considering the relative expression ratios of the respective genes of M plants when calculated using the respective values of NM plants as the control, the expression of *ZmSULTR1.2a* was significantly enhanced after 60 days of S deprivation in M plants, whilst it was strongly downregulated 48 h after the addition of sulfur to the growing medium. Similarly, *ZmSULTR2.1* was upregulated during the long sulfur-deprived period and downregulated immediately after S feeding. The expression levels of *ZmSULTR3.5* were downregulated in the roots of M plants both before (day 60) as well as after (days 61 and 62) sulfur supply. The respective expression of the gene encoding for the vacuolar exporter was strongly upregulated 24 h after the supply of sulfate (Table 2).

### 2.3. Sulfate Transport in Leaves

**Figure 3:**
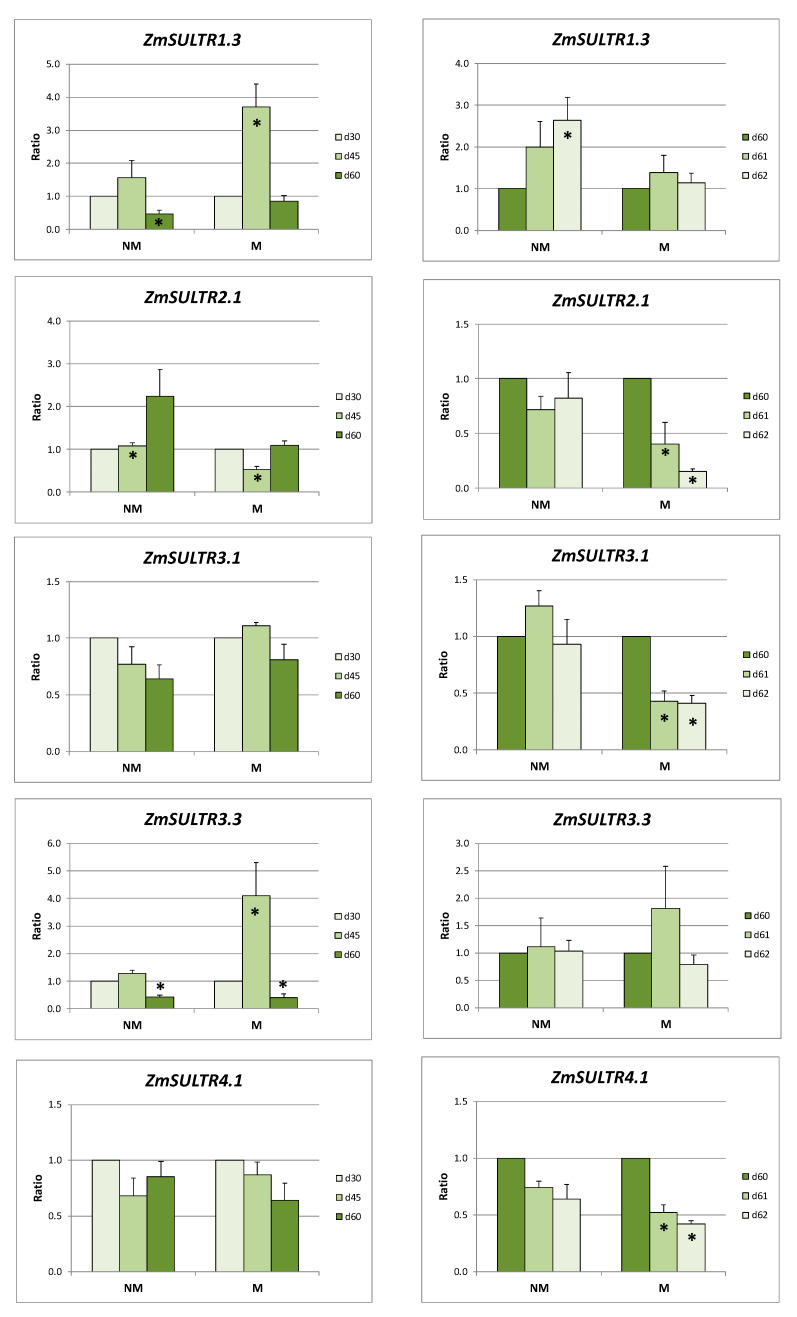
Relative expression ratios (the mean of six biological replicates ± SE) of the sulfate transporters in the leaves of non-mycorrhizal (NM) and mycorrhizal (M) maize plants before (left column) and after (right column) the supply of sulfate. d30/d45/d60/d61/d62: days after sowing. Significant differences (*p* < 0.05) between the samples and the respective controls are represented by an asterisk (*).

A strong overexpression of *ZmSULTR2.1* was observed in the leaves of NM plants at day 60 of the sulfur-deficient period of time. On the contrary, *ZmSULTR1.3* and *ZmSULTR3.3* were strongly downregulated at day 60, whilst the expression levels of *ZmSULTR3.1* and *ZmSULTR4.1* remained constant. In the leaves of M plants, *ZmSULTR1.3* and *ZmSULTR3.3* were transiently upregulated at day 45, while *ZmSULTR2.1* was downregulated at the same day. The expression levels of *ZmSULTR3.1* and *ZmSULTR4.1* also remained unaffected in the leaves of M plants during the prolonged sulfur deprivation period (Figure 3).

Sulfate supply to NM plants resulted in the overexpression of *ZmSULTR1.3*, whereas the expression ratios of all the other genes remained unchanged. In the leaves of M plants, the addition of sulfate induced downregulation of *ZmSULTR2.1*, *ZmSULTR3.1*, and *ZmSULTR4.1*, whilst the *ZmSULTR1.3* and *ZmSULTR3.3* expression levels remained stable (Figure 3).

The relative expression ratios of all the respective genes in the leaves of M plants, when calculated using the respective values of NM plants as the control, showed an increase during the long S deprivation period. The supply of plants with sulfate generally resulted in downregulation (for *ZmSULTR1.3*, *ZmSULTR2.1*, and *ZmSULTR3.3*) or no response (for *ZmSULTR3.1* and *ZmSULTR4.1*) (Table 2).

### 2.4. Sulfate Assimilation in Leaves and Roots

**Figure 4:**
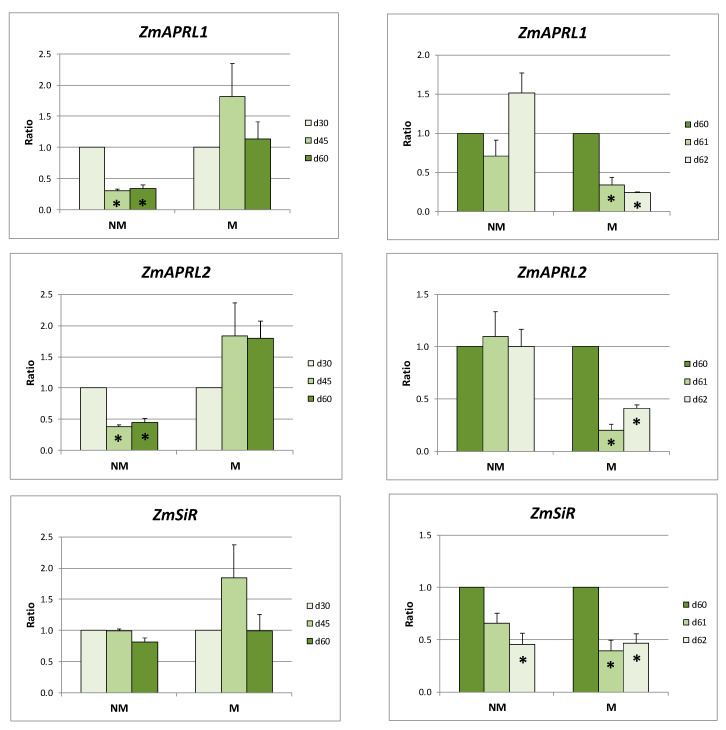
Relative expression ratios (the mean of six biological replicates ± SE) of *ZmAPRIL1, ZmAPRL2*, and *ZmSiR* in the leaves of non-mycorrhizal (NM) and mycorrhizal (M) maize plants before (left column) and after (right column) the supply of sulfate. d30/d45/d60/d61/d62: days after sowing. Significant differences (*p* < 0.05) between the samples and the respective controls are represented by an asterisk (*).

The *ZmAPRL1* and *ZmAPRL2* expression levels in the leaves of NM plants were strongly decreased both at days 45 and 60. On the contrary, the expression levels of these genes remained stable in the leaves of M plants at the same days. The exact opposite results were observed in the leaves of NM and M plants after the sulfate supply: the expression ratios of the two *ZmAPRL* genes remained unaffected in NM plants, whereas they were strongly downregulated in M plants. The expression of *ZmSiR* presented an identical response between NM and M plants: stable during the long sulfur-deficient period of time and reduced after the provision of sulfate (Figure 4).

**Figure 5:**
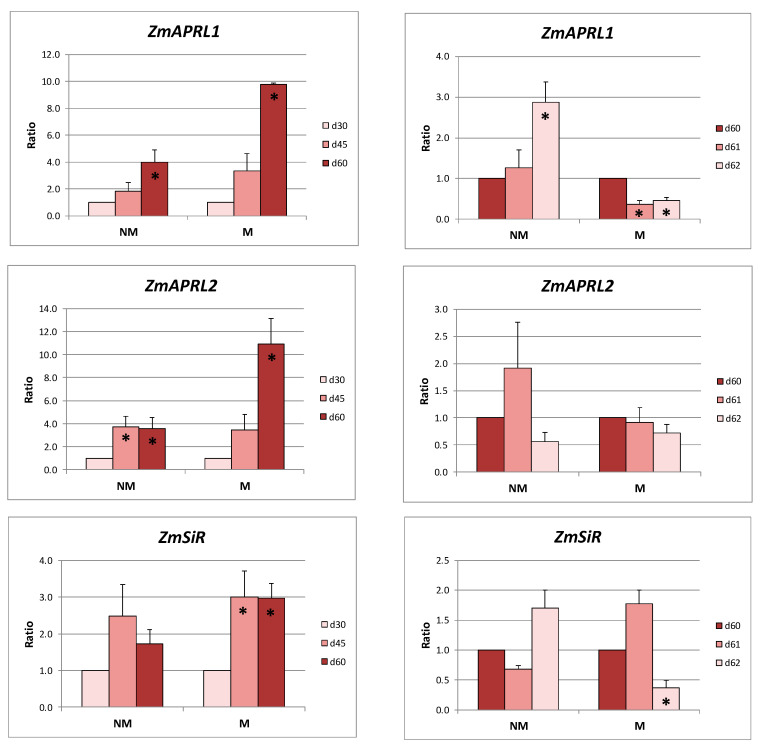
Relative expression ratios (the mean of six biological replicates ± SE) of *ZmAPRIL1, ZmAPRL2*, and *ZmSiR* in the roots of non-mycorrhizal (NM) and mycorrhizal (M) maize plants before (left column) and after (right column) the supply of sulfate. d30/d45/d60/d61/d62: days after sowing. Significant differences (*p* < 0.05) between the samples and the respective controls are represented by an asterisk (*).

Respectively, *ZmAPRL1* and *ZmAPRL2* were strongly overexpressed in the roots of both NM and M plants during the long-term sulfur-deficient period of time. On the contrary, *ZmSiR* expression remained stable in roots of NM plants and increased only in M plants. The addition of sulfate resulted in an overexpression of *ZmAPRL1* in the roots of NM plants and a downregulation of *ZmAPRL1* and *ZmSiR* in the roots of M plants, whilst no statistically significant changes were observed in the expression levels of all other genes and cases (Figure 5).

As far as it concerns the relative expression ratios of the respective genes of M plants when calculated using the respective values of NM plants as the control, both *ZmAPRL1* and *ZmAPRL2* were upregulated in the leaves as well as in the roots of M plants during the 60 days of S deprivation. The addition of sulfate resulted in downregulation of the respective genes. On the contrary, expression levels of *ZmSiR* remained constant in the leaves both before and after the addition of sulfate. The respective levels in the roots of M plants were differentially regulated throughout the treatments (Table 2).

## 3. Discussion

### 3.1. Sulfate Uptake from the Rhizosphere

High-affinity sulfate transporters AtSULTR1.1 and AtSULTR1.2 are the most well-characterized members in the plant SULTR gene family [2,3,4,5,6,7]. Both transporters are responsible for sulfate uptake from the soil whilst being predominantly expressed in root hairs and the rhizodermis, as well as in root cortical cells. It was observed that both mRNA and protein levels of AtSULTR1.1 and AtSULTR1.2 were regulated by sulfur nutrition, where increasing levels coincided with limited supply of sulfur. Among those two high-affinity sulfate transporters, there is strong evidence suggesting that the predominant one is AtSULTR1.2 [2,6,21,22]. This fact may not have favored the existence of a putative SULTR1.1 homolog in maize. Instead, in maize, the SULTR1.2 type is duplicated into SULTR1.2a and SULTR1.2b, whilst only transcripts of *ZmSULTR1.2a* are present in roots, suggesting that this is the major high-affinity sulfate uptake component in maize roots.

As it was expected, prolonged sulfur deprivation resulted in overexpression of the gene encoding putative sulfate transporter ZmSULTR1.2a in roots of both NM and M plants (Figure 2). Especially at day 60, the expression ratio of this gene was higher in M plants compared to NM ones, indicating an increased need of M plants for sulfate uptake (Table 1).

Sulfate supply resulted in a differentiated response in NM and M plants. The expression ratio of *ZmSULTR1.2a* was enhanced in NM plants, whilst it was decreased in M plants (Figure 2). This overexpression in the case of NM plants was indicative of the need to synthesize the precursor of DMA, i.e., methionine, since NM plants were under the Fe-limitation status. On the contrary, since in M plants the pathway of DMA biosynthesis was strongly downregulated [17,23], it is hypothesized that M plants did not exhibit the same need.

### 3.2. Sulfate Distribution in Tissues and Organs

Following the sulfate uptake from the rhizosphere, there are two sulfate transporters which are considered to mainly contribute to the efficient sulfate transport and distribution in tissues and organs: SULTR2.1 and SULTR1.3. *AtSULTR2.1* is expressed in vascular tissues of roots and shoots. It possibly contributes to absorption of sulfate from the apoplast of the central cylinder, resulting in increased concentration of sulfate in xylem parenchyma cells before xylem loading, and it is thus considered to play a significant role in controlling the source-to-sink translocation of sulfate. Especially in roots, enhanced SULTR2.1 transcript levels in xylem parenchyma cells contributes to the increase in the root-to-shoot sulfate transport through increased xylem loading of sulfate. SULTR1.3 is a high-affinity transporter localized in phloem companion cells and important for the source–sink redistribution of sulfate. It is thought to contribute to sulfate unloading from the phloem and its mRNA levels increase under low-sulfur conditions [2,24].

Prolonged sulfur deprivation resulted in overexpression of *ZmSULTR2.1* in roots of both NM and M plants (Figure 2). Especially at days 45 and 60, the expression ratio of this gene was higher in M plants compared to NM ones (Table 2), indicating an enhanced trend for sulfate translocation from roots to sink leaves in M plants. Moving to the aerial plant parts, it is evident that there was a differentiated response of sulfate transport between NM and M plants. *ZmSULTR1.3* was downregulated and *ZmSULTR2.1* was up-regulated in NM plants, whilst M plants have had an opposite response (Figure 3). Downregulation of *ZmSULTR1.3* in NM plants together with the corresponding upregulation of *ZmSULTR2.1* indicates that less sulfate was unloaded from the phloem. On the contrary, in leaves of M plants, downregulation of *ZmSULTR2.*1 together with overexpression of *ZmSULTR1.3* pointed towards enhanced sulfate unloading to leaves. This fact coincided with the aforementioned enhanced trend for sulfate translocation from roots to leaves of M plants.

Sulfate supply resulted in downregulation of *ZmSULTR2.1* in roots and leaves of NM and M plants. On the other hand, the expression ratio of *ZmSULTR1.3* remained stable in the leaves of M plants and increased in NM plants. This response of *ZmSULTR1.3* indicates the efficient transport and phloem unloading to leaves, according to differentiated needs of NM and M plants. 

### 3.3. Intracellular Transport of Sulfate

After entering the cell, sulfate is either metabolized in the cytosol and plastids or stored in vacuoles. Transport systems facilitating import of sulfate to vacuoles are yet unknown. Sulfate transporters localized at the tonoplast membrane mediate the efficient efflux of sulfate from the vacuoles. In *Arabidopsis thaliana*, the expression profile patterns of *AtSULTR4.1* and *AtSULTR4.2* reveal a strong overexpression under sulfur deprivation, whilst their transcripts are mostly present in the pericycle and xylem parenchyma root cells. These characteristics are suggested to be important for increasing the flux of sulfate directed towards xylem loading in roots, especially under sulfur-deficient conditions [2,10].

During the long S-deprived period, *ZmSULTR4.1* was upregulated in the roots of both NM and M plants, following the demand of the plants for sulfate transportation to the leaves. During the same period of time, the respective expression ratios were stable in leaves of all plants (Figure 2 and Figure 3). The supply of sulfate resulted in a transient dramatic increase of the expression ratio of this gene in M plants compared to NM ones at day 61 (Table 2). Massive sulfate import to the roots resulted in a massive vacuole efflux in the roots of M plants for efficient redistribution and root-to-shoot transportation.

The fact that in M plants downregulation of *ZmSULTR2.1* together with overexpression of *ZmSULTR1.3* pointed towards enhanced sulfate unloading to leaves is in agreement with the fact that during the same period of time, overexpression of *ZmSULTR3.3* was observed in M plants, which is believed to be a chloroplast-localized transporter in maize. Group 3 sulfate transporters are all localized on the plastid envelope membrane and believed to contribute to plastidic sulfate import [8,9]. The expression ratio of *ZmSULTR3.3* in NM plants was decreased, probably due to a smaller amount of sulfate which unloaded from the phloem.

On the other hand, the expression ratios of *ZmSULTR3.5* in roots and *ZmSUTR3.1* in roots and leaves remained more or less stable both before and after sulfate supply in both NM and M plants, due to the fact that plastids are the exclusive site for de novo reduction of sulfate to cysteine and thus transport of sulfate into chloroplasts is essential for plants. 

However, when considering the respective expression ratios of M plants compared to NM ones, it is evident that transcripts of *ZmSULTR3.1* were overaccumulated in roots of M plants since day 45 of the treatment (Table 2). According to [17], mycorrhizal symbiosis had already been established by this day. During the arbuscule development, the root cortical cells’ plasma membrane undergoes reorganization and it expands to envelop the hyphal branches to form the so called periarbuscular membrane, which separates the fungal hyphae from the host cytoplasm. Moreover, *ZmSULTR3.1* shares high similarity to the symbiosome membrane-localizing sulfate transporter SST1 from *Lotus japonicus*. It is thus suggested that in maize, *ZmSULTR3.1* is the sulfate transporter localized in the periarbuscular membrane contributing to the exchange of sulfate with the fungus.

### 3.4. Sulfate Assimilation 

The observed expression patterns of the genes related to sulfate uptake and transport during the long S-deprived period of time in roots coincided with overexpression of the two putative APS reductases in both NM and M plants (Figure 5). During the same period of time, the expression ratios of these two genes remained stable in the leaves of M plants, where enhanced sulfate unloading, adequate to support sulfur assimilation, seemed to take place. Moreover, overexpression of both APR isoforms before sulfate supply in the leaves of mycorrhizal plants indicates that the mycorrhizal plants may have enhanced sulfur demand in order to sustain higher photosynthetic rates. The opposite was observed in the leaves of NM plants: downregulation of *ZmSULTR1.3* together with upregulation of *ZmSULTR2.1*, indicative responses of less sulfate unloaded from the phloem, coincided with the reduced expression ratios of the two maize APRs in leaves (Figure 3 and Figure 4).

Sulfate supply seemed to not have any impact on the expression ratios of the two putative APRs in roots of both NM and M plants, with the exception of *ZmAPRL1* in NM plants at day 62. In leaves, downregulation was observed in M plants, whilst the respective expression ratios in NM plants were not affected by the treatment. These results are consistent with the hypothesis that NM plants have a higher demand for organic sulfur in order to support the strategy II Fe acquisition pathway, which resulted in a more or less stable expression of *ZmAPRL1* and *ZmAPRL2* in a demand-driven manner.

## 4. Materials and Methods 

### 4.1. Plant Material and Growth Conditions 

Maize (*Zea mays* L., “Cisko”, Syngenta Hellas) seeds were placed to germinate in a dark and high-humidity environment for 4 days. Then, the maize seedlings were transferred to culture boxes containing aerated distilled water for the next 4 days. Eight days after sowing, the mesocotyl and the embryonic root system were detached from the seedlings and those seedlings were then supplied for 2 more days with an aerated nutrient solution which contained a low P concentration (10 μΜ) but no Fe or S. Ten-day-old maize seedlings carrying only nodal roots (without their below-crown-parts) were transferred to pots with sterile river sand. The amount of 500 mg of the sparingly soluble FePO4 was added to each pot, whilst for the mycorrhizal plants, 300 mg of the *Rhizophagus irregularis* inoculum (synonym: *Glomus irregulare* DAOM197198, SYMPLANTA-001 standard grade, Symplanta) were added to the respective pots. One maize plant was grown in each pot and six pots per treatment and sampling were used. The pots were placed completely randomly in a controlled environment [17] and were rotated six times throughout the experiment. Mycorrhizal (M) and non-mycorrhizal (NM) plants were watered two times a week with an S- and Fe-deficient nutrient solution until day 60 from sowing. On day 60 from sowing, sulfur was provided to the plants in the form of sulfate. The S- and Fe-deficient nutrient solution contained 5 mM KNO_3_, 10 μM KH_2_PO_4_, 2 mM Mg(NO_3_)_2_ 6H_2_O, 4 mM Ca(NO_3_)_2_ 4H_2_O, 0.86 mM CaCl_2_ 2H_2_O, 0.9 μM ZnCl_2_, 30 μM H_3_BO_3_, 0.9 μM CuCl_2_ 2H_2_O, 0.5 μM MoO_3_ 85%, and 20 μM MnCl_2_ 4H_2_O. The nutrient solution supplied to the plants on day 60 contained 5 mM KNO_3_, 10 μM KH_2_PO_4_, 2 mM Mg(NO_3_)_2_ 6H_2_O, 2.5 mM CaSO_4_ 2H_2_O, 1 mM MgSO_4_ 7H_2_O, 4 mM Ca(NO_3_)_2_ 4H_2_O, 0.9 μM ZnCl_2_, 30 μM H_3_BO_3_, 0.9 μM CuCl_2_ 2H_2_O, 0.5 μM MoO_3_ 85%, and 20 μM MnCl_2_ 4H_2_O. All the chemicals were purchased from Sigma-Aldrich (Saint Louis, MO, USA).

### 4.2. Plant Samplings

Samplings were performed both before and after sulfur supply. During the long-term sulfur deprivation, samplings took place on days 30, 45, and 60 after sowing. The responses of the plants to sulfur supply were recorded 24 and 48 h after sulfur supply (samplings on days 61 and 62). All the samplings were performed 3 h after the onset of light. Lateral roots, as well as two young expanding leaves, were immediately frozen in liquid nitrogen and stored at –80 °C until use. In each experiment, plant material from three biological replicates per treatment and sampling day was used [17].

### 4.3. In Silico and Gene Expression Analysis

All sequence alignments were performed using T-Coffee (http://tcoffee.crg.cat/), and phylogenetic trees were constructed with MEGA X. The pattern of gene expression for the selected genes was studied by RT-PCR as already described [17]. The oligonucleotide primers used for this analysis are listed in Table 3 (primer synthesis was performed by Eurofins Genomics). Recombinant DNase I (RNase-free) and reverse transcriptase PrimeScript RT reagent (Perfect Real Time) were provided by Takara Bio Inc. The KAPA SYBR FAST Master Mix was provided by KAPA Biosystems. RT-PCR was performed in an MxPro Mx3005P thermocycler (Stratagene, La Jolla, CA 92037USA). The gene of ubiquitin was used as an internal control and the target was detected by the following primers pair: *ZmUBQ* (MaizeGDB Gene ID: GRMZM2G431821): forward 5′-TGTCTTCATGG CCAACCACT-3′ and reverse 5′-GCTTGATAGGTAGGC GGGTG-3′. 

The LinRegPCR software was exploited to determine efficiency of each RT-PCR [25]. In order to calculate the relative expression ratios of the genes studied, the following mathematical formula was utilized [26]. Ubiquitin (*ZmUBQ*) was selected as the reference:

(1) $$ratio=\frac{E{\mathrm{target}}^{\Delta CP\mathrm{target}\left(control-sample\right)}}{E{\mathrm{ref}}^{\Delta CP\mathrm{ref}\left(control-sample\right)}}$$

As controls for both treatments (NM or M), we selected the samples of day 30 (for the samplings prior to sulfur supply) or day 60 (for the samplings after sulfur supply), unless otherwise specified. 

### 4.4. Statistical Analysis

This experiment was carried out twice under identical conditions and during two distinct time periods: autumn 2013 and spring 2014. In order to determine the significance of differences between samplings, the data were analyzed using the *t*-test variance analysis with two-tailed distribution and two-sample unequal variance in Microsoft Excel. 

## 5. Conclusions

Mycorrhizal plants presented an enhanced tendency for sulfate translocation from roots to shoots, as well as for sulfate unloading to young leaves during S deprivation. These observations coincided with the increased trend of sulfate transport to chloroplasts and increased sulfate assimilation in the leaves of mycorrhizal plants, indicating a higher demand for organic sulfur in their leaves in order to sustain higher photosynthetic rates towards efficient feeding of their symbiotic partner. On the contrary, non-mycorrhizal plants seemed to restrict sulfate translocation outside the roots, whilst at the same time presented an increased trend for sulfate assimilation in their roots, indicating a higher demand for organic sulfur in their roots, probably in order to support the strategy II Fe acquisition pathway and DMA biosynthesis.

## Figures and Tables

**Table 1:** Putative sulfate transporters, adenosine 5’-phosphosulfate reductases (APRs) and sulfite reductase (SiR) of *Zea mays*.

Gene	Maize Genome Database Gene ID		Chromosome
	B73 RefGen_v3	Zm-B73-REFERENCE-GRAMENE-4.0	
*ZmSULTR1.2a*	GRMZM2G159632	Zm00001d028162	1
*ZmSULTR1.2b*	GRMZM2G342907	Zm00001d048189	9
*ZmSULTR1.3*	GRMZM2G080178	Zm00001d050283	4
*ZmSULTR2.1*	GRMZM2G042171	Zm00001d028164	1
*ZmSULTR3.1*	GRMZM2G154211	Zm00001d027749	1
*ZmSULTR3.3*	GRMZM2G395114	Zm00001d002038	2
*ZmSULTR3.4*	GRMZM2G444801	Zm00001d000204	9
*ZmSULTR3.5*	GRMZM2G158013	Zm00001d043614	3
*ZmSULTR4.1*	GRMZM2G068212	Zm00001d005257	2
*ZmAPRL1*	AC189750.4_FG004	Zm00001d021596	7
*ZmAPRL2*	GRMZM2G087254	Zm00001d006467	2
*ZmSiR*	GRMZM2G090338	Zm00001d038625	6

**Table 2:** Relative expression ratios (the mean of six biological replicates ± SE) of the sulfate transporter and assimilation genes in leaves and roots of mycorrhizal plants. For these calculations, non-mycorrhizal plants were selected as the respective control. Significant differences (*p* < 0.05) are depicted with a highlighted table cell. When upregulation of a gene was observed, the respective table cell is dark grey, whilst a light grey cell represents downregulation of the gene. Samplings before sulfur supply: days 30, 45, and 60. Samplings after sulfur supply: days 61 and 62.

		Days after Sowing				
	Gene	30	45	60	61	62
**Leaves**	*ZmSiR*	0.89 ± 0.15	1.49 ± 0.31	1.35 ± 0.26	0.85 ± 0.11	1.52 ± 0.30
	*ZmAPRL2*	0.70 ± 0.10	1.87 ± 0.18	2.48 ± 0.58	1.04 ± 0.21	1.07 ± 0.17
	*ZmAPRL1*	0.82 ± 0.24	2.30 ± 0.42	2.22 ± 0.39	1.33 ± 0.28	0.38 ± 0.09
	*ZmSULTR3.3*	1.01 ± 0.17	3.02 ± 0.33	0.91 ± 0.13	1.27 ± 0.35	0.44 ± 0.11
	*ZmSULTR3.1*	1.19 ± 0.31	1.81 ± 0.14	1.62 ± 0.57	0.90 ± 0.31	1.07 ± 0.19
	*ZmSULTR4.1*	1.89 ± 0.22	2.28 ± 0.38	1.34 ± 0.27	1.13 ± 0.14	1.00 ± 0.13
	*ZmSULTR2.1*	2.84 ± 0.47	1.19 ± 0.29	2.24 ± 0.36	2.27 ± 0.76	0.64 ± 0.08
	*ZmSULTR1.3*	1.30 ± 0.25	2.74 ± 0.16	1.97 ± 0.24	1.22 ± 0.20	0.68 ± 0.15
**Roots**	*ZmSiR*	1.39 ± 0.29	1.79 ± 0.14	1.89 ± 0.22	3.21 ± 0.84	0.33 ± 0.07
	*ZmAPRL2*	0.65 ± 0.11	1.18 ± 0.30	2.97 ± 0.28	1.21 ± 0.23	0.56 ± 0.14
	*ZmAPRL1*	0.70 ± 0.19	1.54 ± 0.41	3.40 ± 0.49	0.80 ± 0.25	0.54 ± 0.19
	*ZmSULTR3.6*	1.54 ± 0.32	1.00 ± 0.16	0.53 ± 0.18	0.64 ± 0.15	0.43 ± 0.12
	*ZmSULTR3.1*	0.45 ± 0.21	2.49 ± 0.67	1.85 ± 0.32	1.82 ± 0.17	4.07 ± 1.24
	*ZmSULTR4.1*	1.97 ± 0.58	1.35 ± 0.37	1.68 ± 0.13	34.80 ± 4.90	0.40 ± 0.08
	*ZmSULTR2.1*	1.00 ± 0.16	3.51 ± 0.96	1.94 ± 0.33	0.66 ± 0.11	1.26 ± 0.14
	*ZmSULTR1.2a*	1.00 ± 0.26	1.12 ± 0.21	3.17 ± 0.74	1.21 ± 0.30	0.09 ± 0.02

**Table 3:** The oligonucleotide primers used for this analysis.

	Primer Sequences Used in RT-PCR (5’ to 3’)	
Gene	Forward	Reverse
*ZmSULTR1.2a*	AAGATATTCCTCACGGTCGGC	AGACTTCTGGCTCGTACTG
*ZmSULTR1.3*	CTCCAAAAGCGAGGCATTCAG	GCGACCGTGAGGAAGATATGAC
*ZmSULTR2.1*	CGGGATGGAAGGCAGTTCA	TCCACCGCTTCACCTACTGT
*ZmSULTR3.1*	CCGGTCTGACGTAACCAGTC	AATTCGTTTTTCCCGCGACG
*ZmSULTR3.3*	ATGCAGATCACAAGGCCGAA	TGGCGAAGTTTATCGGTGCT
*ZmSULTR3.5*	CAGGCAGGAAAGCAAGCATC	GGAGAGACCAGATGCCACAC
*ZmSULTR4.1*	CGTGTTGACCGATTCTGTTTCG	AAGGTTTTGCCGGAGCAGT
*ZmAPRL1*	GACACAGGAAGGAACGAGGG	CTCAGCTACCAAGCACAGGG
*ZmAPRL2*	CCATCTAGGTGCGCTGTACG	AAATCTCTCCCTCAGCTGCC
*ZmSiR*	GCTGAACGGGGGATCTTACC	CTAGCGCATCCATTAGGGCA

## Abbreviations

NM	Non-mycorrhizal
M	Mycorrhizal
SULTR	Sulfate Transporter
APS	Adenosine 5’-phosphosulfate
APR	APS reductase
SiR	Sulfite reductase
DMA	Deoxymugineic acid
